# Influence of Cleats-Surface Interaction on the Performance and Risk of Injury in Soccer: A Systematic Review

**DOI:** 10.1155/2017/1305479

**Published:** 2017-06-08

**Authors:** Diogo C. F. Silva, Rubim Santos, João Paulo Vilas-Boas, Rui Macedo, António Mesquita Montes, Andreia S. P. Sousa

**Affiliations:** ^1^Área Científica de Ciências Funcionais, Escola Superior de Saúde do Porto, Instituto Politécnico do Porto, Centro de Estudos de Movimento e Atividade Humana, Rua Dr. António Bernardino de Almeida 400, 4200-072 Porto, Portugal; ^2^Área Científica de Física, Escola Superior de Saúde do Porto, Instituto Politécnico do Porto, Centro de Estudos de Movimento e Atividade Humana, Rua Dr. António Bernardino de Almeida 400, 4200-072 Porto, Portugal; ^3^Faculdade de Desporto, CIFI2D, Universidade de Desporto e Laboratório de Biomecânica do Porto, Universidade do Porto, Porto, Portugal; ^4^Área Científica de Fisioterapia, Escola Superior de Saúde do Porto, Instituto Politécnico do Porto, Centro de Estudos de Movimento e Atividade Humana, Rua Dr. António Bernardino de Almeida 400, 4200-072 Porto, Portugal

## Abstract

**Objective:**

To review the influence of cleats-surface interaction on the performance and risk of injury in soccer athletes.

**Design:**

Systematic review.

**Data Sources:**

Scopus, Web of science, PubMed, and B-on.

**Eligibility Criteria:**

Full experimental and original papers, written in English that studied the influence of soccer cleats on sports performance and injury risk in artificial or natural grass.

**Results:**

Twenty-three articles were included in this review: nine related to performance and fourteen to injury risk. On artificial grass, the soft ground model on dry and wet conditions and the turf model in wet conditions are related to worse performance. Compared to rounded studs, bladed ones improve performance during changes of directions in both natural and synthetic grass. Cleat models presenting better traction on the stance leg improve ball velocity while those presenting a homogeneous pressure across the foot promote better kicking accuracy. Bladed studs can be considered less secure by increasing plantar pressure on lateral border. The turf model decrease peak plantar pressure compared to other studded models.

**Conclusion:**

The *soft ground* model provides lower performance especially on artificial grass, while the turf model provides a high protective effect in both fields.

## 1. Introduction

Soccer is the most practiced and most popular sport worldwide [1]. This sport is followed by millions of people around the globe, mobilizing people to the stadium, to watch games on TV/internet, and to listen via radio. Its popularity turned it into an industry where the sports scores and goals achieved are of the utmost importance [2]. Therefore, the importance of this sport supports the need of looking for strategies to improve athletes' performance, but also to prevent sports-related injuries. This will allow players to provide the best possible spectacle to his fans, while improve their carriers and clubs [3].

Several adaptations have been introduced in soccer along the years. The increasing use of artificial grass field [4, 5] and changes in format and materials used in soccer cleat are examples of this adaptation [6, 7]. These changes agree with the increased importance attributed to the cleat-surface interaction in both performance and the injury risk. The adequacy of soccer footwear to the kind of field seems to have a determinant role in both [7–10]. Several research studies have been developed regarding this area. However, there is no broad consensus as to the adequacy of the kind of the cleat to the respective field to fulfill the requirements of performance and injury risk. The different study methodologies and the funding from shoe or turf companies can possibly contribute to this divergence [11–18]. The lack of consensus in this topic has been recently demonstrated in a qualitative review [9]. The authors did not conclude about the best cleat to reduce the injury risk and to improve performance. Inside of this, the authors have made several conclusions as to general aspects of shoe-surface interaction. This difficulty can be based on the large variability of sports modality englobed in the review [9]. Because each modality has specific sports gestures that impose different demands on cleat-surface interaction, as well as different rules, each sports modality should be considered separately [3, 19, 20].

The cleats have been considered the most important soccer tool, playing a crucial role in the athletes' performance [7, 10]. Its structure can be divided into two main parts, the upper portion, composed by leather or synthetic material, and the sole. The structure of the sole depends on the pitch and is adjusted to provide a good contact with the ground. The studs should provide enough traction to prevent slipping or sliding, which can result in overstretch or tear injuries, but should facilitate sudden change of directions [6]. The distribution pattern and geometry of studs vary widely between models and manufacturers [3]. Currently, there are basically five types of soles: *turf* (TF), *artificial grass* (AG), *hard ground* (HG), *firm ground* (FG), and *soft ground* (SG) [6, 16]. According to the manufacturers, the TF and AG models are suitable for artificial fields and *HG* model for hard, natural, or dirt soccer fields. The *FG* model is indicated for natural grass in good conditions, while the SG to very muddy or wet natural fields. The classification of these models depends on the size, number, distribution, and type of studs. Thus, the first model (TF) presents the highest number of studs, but also the smaller ones. The other models present a progressive decrease in the number of studs and an increase in its size [16]. Normally, the SG model is characterized by rigid plastic soles and only six aluminum studs. In the TF model, the sole and studs are usually composed by rubber while the AG, HG, and FG models present rigid soles and studs, usually made of plastic [6]. Another feature that varies constantly is the stud geometry (cylindrical, conical, prismatic, and bladed) [13], and for this reason, several studies have questioned if the increased traction promoted by bladed studs improves performance during sudden changes of direction or, on the contrary, could increase the risk of injury [11, 12, 14–16, 18, 21]. The cleats' characteristics are summarized in Table 1.

With the increasing number of models available on the market, it becomes important to review the influence of cleat-surface interaction on athletes' performance and injury risk to identify the cleat that better responds to the need of increased performance and reduced injury risk.

## 2. Methods

### 2.1. Research Question

The two main research questions in this study were as follows:

1. Which model of soccer cleats promotes a better performance in artificial and natural grass?
2. Which model of soccer decreases the risk of injury in artificial and natural grass?

### 2.2. Search Strategy

The literature search included only the period from 2000 until 2016 on the following databases: Scopus, Web of science, PubMed, and B-on (Table 2).

The following search term combinations were used in all databases: soccer shoes; soccer boots; soccer cleats; soccer studs; soccer footwear; shoe-surface interface, and shoe-surface interaction. The search terms were limited to titles and abstracts published in academic journals. The reference lists of all studies were also scanned to identify other potential eligible articles. The study was conducted using the systematic review method proposed by the Preferred Reporting Items for Systematic Reviews and Meta-Analysis—PRISMA [22]. The articles included in this review were as follows: (i) experimental and original papers, written in English; (ii) studied soccer cleats' influence on sports performance in artificial or natural grass; (iii) studied soccer cleats' influence on injury risk in artificial or natural grass analysis; (iv) compared more than one cleat model in sport tasks; (v) analyzed young and adult soccer players or used mechanical devices; and (vi) studied soccer players of both genders and all competitive levels. Review articles and those that studied rugby or American football cleats were excluded because the technical gesture and the rules of this sport differ significantly from soccer.

### 2.3. Assessment of Methodologic Quality

The studies included in this systematic review were evaluated using a quality index proposed by Downs and Black [23] and the recommendations of Munn et al. [24]. Studies meeting <60% criteria were considered low quality, 60%–74.9% moderate quality, and >75% high quality. Each author independently performed the quality assessment for each of the included studies. Consensus regarding the quality index score for each study was agreed upon by both authors.

### 2.4. Data Extraction

Data from the included studies was extracted by one reviewer and then checked by a second reviewer using a data extraction table which identified the following: author identification, year of publication, sample, ground and footwear conditions, methods and instruments, variables assessed, and main conclusions regarding the shoe-surface interaction on performance and injury risk.

## 3. Results

**Figure 1:**
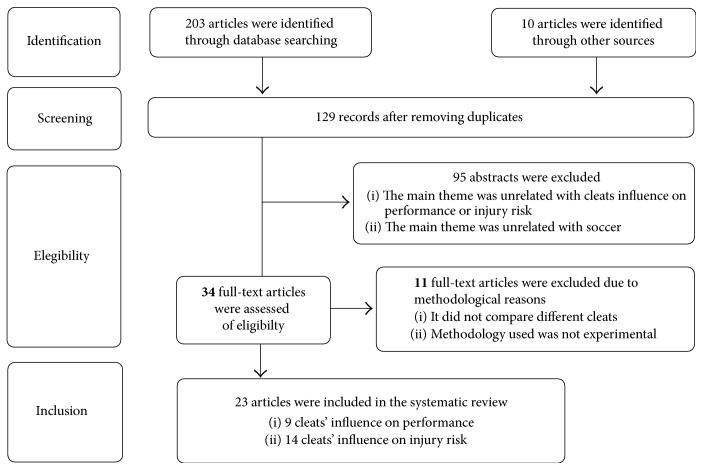
Study selection and inclusion criteria.

The search strategy revealed 213 articles. After an initial review, 84 were rejected as copies of the same paper and 95 were excluded as they were clearly unrelated with the main theme or because the sport studied was not soccer. All remaining articles were then reviewed by two independent reviewers. Consensus was reached, and a total of 23 were included as shown in Figure 1. Nine of them were related to performance and fourteen with injury risk.

### 3.1. Study Design and Sample: Cleat-Surface Interaction on Performance

Most of the studies assessed the traction imposed by different cleat models during sprint or change of direction maneuvers [11, 13–15, 18, 25]. Some studies evaluated other sport performance components, such as kicking velocity [26] and accuracy [27] and the ability to handle a ball [28]. With the exception of two studies that evaluated the cleats on natural and artificial fields [11, 13], the majority included artificial grass field in their set up [14, 15, 18, 25, 26, 28]. The authors that have evaluated the kicking accuracy did not provide information regarding the kind of field in which the tests were performed [27]. Only one study based the results on mechanical simulations [13]. All other studies obtained their results from experienced male soccer players. The sample size ranged from 12 to 52 athletes, with age ranging between 16 and 25 years, the body weight between 67 to 77.5 Kg, and height between 176 and 181 cm [11, 14, 15, 18, 25–28]. In Table 3 are synthetized main features of the studies described.

#### 3.1.1. Synthesis of the Results: Cleat-Surface Interaction on Performance

The findings obtained in artificial grass showed that generally SG models decrease performance [11, 15]; however on wet ground, the TF provides the lowest performance [25]. The results obtained with a specific cleat prototype for artificial grass englobing sole characteristics from the AG and FG models favored performance compared to all other commercialized models [11, 14].

The studies that have addressed specific cleat characteristics demonstrated the following: (i) bladed studs improved performance compared with the elliptical ones [11, 28]; (ii) increased stud height seems to improve performance [11], since the studs can fully penetrate [13]; (iii) models that allowed a more homogeneous pressure across the foot during ball contacts promoted a better accuracy of kicking [27]; (iv) the cleat weight or heel comfort seem to not interfere with performance [11]; and (v) the maximum ball velocity was achieved with cleats that promoted a better traction in the standing limb [26]. However, players can adjust the sport gesture to maintain the desired level of traction in sport tasks [18].

### 3.2. Study Design and Sample: Cleat-Surface Interaction on Injury Risk

Most of the studies stated their conclusion based on dynamic tasks like straight running [21, 29, 30], slalom [21], cutting, and turning maneuvers [16, 31–35]. Only the three most recent studies have incorporated jump [36] or landing tasks with changes of direction [35, 37]. Four studies based their conclusions on peak torque and the translation or rotational stiffness assessed from mechanical simulations [12, 17, 33, 38]. The other studies based their conclusions on plantar pressures [16, 21, 29], the ankle or knee range of movement [29, 34–37], the ground reaction forces [30–32, 34–37], and neuromuscular variables [32, 37] collected from soccer players. Nine articles analyzed the cleats on artificial grass [16, 21, 31–37], one on natural grass [30], three on both fields [12, 17, 38], and one did not provide this information [29]. This last study was the only that assessed young players. The majority of the studies relied on experienced male [21, 30–35, 37] and both gender [16, 36] soccer players. The sample size ranged from 6 to 36 athletes, with age ranging between 8 and 26 years, the body weight between 64 to 85 Kg, and height between 168 and 183 cm. In Table 4 are compiled main features of the studies described.

#### 3.2.1. Synthesis of the Results: Cleat-Surface Interaction on Injury Risk

In artificial grass, the TF model seems to be the best choice to prevent injuries related to repetitive impacts, when compared to FG and HG [16], and probably to reduce the risk of ankle and knee injury in turning movements, when compared to FG and SG models [33]. The increased risk of injury with FG and SG models seem to be explained by an increased and unsuitable traction promoted by these cleats [34]. On another hand and surprisingly, the lower peak of medial ground reaction force demonstrated in SG model when compared with artificial grass studs seem to favor the use of this model [35]. Furthermore, when more specific related injury risk variables were studied (ankle sprain), no differences were observed between different models of cleats (TF, HG, and FG), even after an evertor-oriented fatigue protocol [37].

The studies that have addressed specific cleat characteristics demonstrated the following: (i) the use of cleats without studs (similar to TF model) when compared to cleats with studs could decrease the incidence of calcaneal apophysitis [29] and (ii) bladed studs revealed an increased risk of injury related to higher pressure on the lateral border of the foot when compared to rounded studs [21] and impaired female reception mechanism after a jump [36], but no differences were observed in knee loading [31–33] or in peak torque measured by a mechanical device [17].

Once again, in natural grass fields, the TF model revealed as the best choice when compared to SG model to prevent injuries related to repetitive impacts [30]. Lastly, like it was stated for the performance, the kind of field has an important role in injury risk. When natural and artificial grass was compared, the last one showed a higher peak torque [38], rotational traction [12], and stiffness [17] evaluated by a mechanical testing device.

## 4. Discussion

### 4.1. Research Question 1: How Cleat-Surface Interaction Affects the Performance?

Since 2008, Sterzing and coworkers evaluated various cleat models in two different fields (natural and artificial grass) during different functional tasks like slalom and short straight line acceleration [11, 14, 15, 18, 25], kicking [26, 27], passing, or handling a ball [28].

In general, the model defined by the manufacturers as indicated for artificial grass has been demonstrated to favor performance in this kind of field compared to design model for natural grass (SG) and this is perceived by athletes [11, 15]. The same studies revealed that SG cleats decrease performance in dry or wet artificial grass comparing to the other models probably because this model seems unable to fully penetrate into this ground, causing instability mechanisms [14, 15]. Globally, the athletes' performance seems to be worsened when the stud height is reduced on dry conditions [11], but also on wet conditions, due to a lack of traction [25]. The studs' geometry seems to be an influent factor in performance between different models of cleats. Bladed studs allowed better performance compared with the elliptical in slalom tests [11] and dribbling [28]. The bladed shape of these studs and his orientation to the front may lead to increased traction in mediolateral maneuvers, and this could explain these results. It has also been demonstrated that studs with larger cross section area (not fully penetrated) provide decreased traction, and because of that, it could provide decreased performance [13]. Finally, a prototype cleats' sole, similar to a regular FG outsole at the rearfoot, but with multiple double cylindrical thermoplastic polyurethane elastomers stud elements (DuoCell Techonology) at the forefoot, was demonstrated to be more suitable for artificial fields, compared to three already commercialized models to natural fields [14]. In terms of performance, this prototype enabled the manufacturers to reflect about the ideal model for this type of field.

Studies performed on natural grass revealed that despite not being perceived by the athletes, bladed studs are associated to increased performance compared to the elliptical ones in dry or ice and snow conditions [11]. In this kind of field, the heel contour comfort and weight do not seem to interfere with the performance, at least in short performance tests [11, 26]. However, we do not know if these two characteristics interfere with the performance in real game conditions. In this sense, further studies are required on this topic. Later studies have concluded that in natural grass, only cylindrical and highest studs not fully penetrate the field, which may explain the lack of traction. For this purpose, the conical studs provide better results [13]. Having a lower cross-sectional area, this last stud geometry could have a major role in the degree of cleat penetration on natural grass.

Other performance tests regarding kicking tasks revealed that cleats that promote a good traction on the support leg appear to enhance the speed of the shot, while outsole stiffness does not contribute to increased kick velocity [26]. The stability of the support leg should be highlighted, since it seems to be a key point to improve the performance of the shot. Also, pass assertiveness can be positively influenced by the cleat presenting a more homogeneous pressure distribution between the upper shoe part and the ball [27]. Furthermore, the dribbling capacity and velocity appears to be enhanced by FG bladed model compared to FG round model [28] maybe because the slalom velocity inherent to this task is improved by bladed studs compared to the rounded ones [11].

Artificial grass features numerous characteristics, such as infill particle size, level of compaction, and fiber type; however, only few characteristics have been considered in most of the papers [11-13]. Some of these characteristics have been demonstrated to influence the athletes' performance [18]. McGhie and Ettema [18] evaluated three models of cleats in three artificial grass conditions and have concluded that the pitch with smaller size of artificial grass and less rubber fill imposes more traction than the others. The dry or wet state of the artificial grass is another feature that influences performance. In wet conditions, the running time was increased with the TF model in relation to AG and FG models. The smaller studs founded in the TF model decreases their traction and therefore their performance [25]. The research about this theme has increased along time, especially in artificial grass supporting the growing incentive by FIFA for the use of this type of ground [4, 5].

Despite the high ability of athletes to compensate the different mechanical traction imposed by different cleats during a dynamic task, the findings obtained by the previously mentioned studies (Table 3) demonstrate that the cleat characteristics, together with the kind of field, can determine the effort required for a given performance [15]. In fact, the studies mentioned in this review indicate that SG cleats impair performance, especially on artificial grounds. This model presents high studs, and it does not always allow their full penetration in the field, making traction difficult and worsening the execution of functional speed tests [11, 13–15]. Concerning the studs' geometry, the bladed models could improve performance, compared with the round studs, in slalom movements, whether in dry ground or with ice/snow. This particular model seems to increase the medial-lateral traction facilitating this type of changes of direction [11]. Also, no differences seem to exist between the TF, *HG*, and *FG* models in terms of performance [11, 14, 15, 18], unless the artificial grass is wet, which imposes decreased shuttle run test performance with the TF model [25]. However, these results should be considered with caution, since performance was evaluated only in healthy subjects through velocity in sprints, diverging just in the direction, straight or with direction shifts to 45°, 90°, and 180°, as well as slalom sprint [11, 14, 15, 18, 25]. None of the studies adopted functional tests more close to sport modality, like jumps with sprints that can be influenced by the type of footwear and ground [36]. It should be noted that most of the studies have included male and young adult athletes from lower divisions, or amateurs [11, 14, 15, 18, 25, 27]. Given the increasing popularity of this sport among women, it makes sense to extend this kind of studies also to this population.

### 4.2. Research Question 2: How Cleat-Surface Interaction Affects the Injury Risk?

Various cleat models have been evaluated since 2002 in both natural and artificial fields during different functional tasks (straight running, slalom, cutting and turning maneuvers, and landing after jumps). Unlike in the previous research question, this interaction was investigated not only in male athletes [16, 21, 30–37] but also in female adults [16, 36] and young athletes [29]. On the other hand, to answer the present research question, some articles used mechanical instruments [12, 17, 33, 38].

For a better understanding, the results will be discussed considering the variables/instruments used to measure the injury risk of the different cleat models. First of all, it is important to highlight that most of authors used more than one instrument, combining, frequently, the use of motion cameras systems and force platforms [31–37]. Whether on natural or on artificial fields, it has been demonstrated that adult players using aluminum studs (SG) present an increased vertical ground reaction forces which could be associated with injuries caused by repeated impacts [30, 34]. These findings seem not support the use of SG in hard grounds. The TF model presenting increased compliance [6] seems to be more indicated to prevent this kind of injuries [30]. Furthermore, cleats with removed studs increase the risk of slipping whereas the SG sole configuration with aluminum studs induce high loads on the player [34]. However, surprisingly, during 180° cut movements in artificial grass, aluminum studs seem to produce the lowest peak medial ground reaction forces compared to artificial grass studs and nonstudded running shoe [35]. These findings could be related to the insufficient penetration showed by this model in artificial grass that could have induced a feeling of instability [13, 15] and led players to perform the task slowly. Apparently, there are no major differences between the TF, HG, and FG models regarding kinetic (loading rate of ground reaction forces) and kinematic data (eversion/inversion range of movement, COP displacement, and velocity) following jump with changes of direction. This is true even when the players were under a fatigue protocol for the main lateral ankle stabilizers. This conclusion must be considered carefully, since the fatigue protocol was applied to a small and specific muscle group of the ankle and the sample was composed by healthy athletes (without ankle sprain history). The nonexistence of differences could be due to the great capacity of healthy athletes to compensate small differences between models [37]. Additionally, during running and cutting maneuvers, no differences in ankle [33] and knee [31, 32] joint moments between FG (rounded and bladed) and SG (rounded and bladed) models were showed. Nevertheless, the rounded FG model when compared with the bladed FG appears to potentiate the quadriceps femoris activation, which can be associated with an increased internal load on the anterior cruciate ligament [32]. This finding should be interpreted with caution because of the small sample size. Lastly, it should be noted that the only study that assessed a pure jump task showed that more rigid shoes (bladed cleats compared to the running shoes or TF model) seem to impair the landing mechanism both in male and female players. Special attention should be given to this finding since female players present increased risk of lower limb injury [36]. A study involving young soccer population demonstrated that cleats with studs lead to a significant increase in dorsiflexion during the middle phase of support while running and a consequent increased pressure on the growth center of the calcaneus. Therefore, the high incidence of calcaneal apophysitis and the use of shoes with studs in young populations might be related [29]. This article has a great importance because it encourages the young soccer players to make the best choices regarding the choice of footwear for different fields. In the education process of the athletes, it makes sense to start with the youngest.

Plantar pressure distribution and neuromuscular variables could give important insights regarding the risk of injury. However, few studies have addressed these variables. The TF model appears to be the only cleat that decreases the force and pressure beneath the metatarsal heads and, for that reason, could possibly minimize metatarsal injury risk [16]. The bladed studs imposes increased plantar pressure on the lateral border of the shoe, while the model with rounded studs can be considered more secure since it leads to pressure distributions that mimic the normal plantar pressure profile [21]. Neuromuscular variables, such as activation time, were addressed in one study only. Despite its importance for the risk of injury assessment, no differences were observed in the peroneal activation time between TF, HG, and FG models, even under fatigue. These results should be considered with caution since it can be questioned if the isolated fatigue of the peroneal muscles could be sufficient to impair the postural control mechanisms [37].

Some authors encouraged the study of cleat-surface interaction using sporting gestures performed in place of practice/game [39]; however, some interesting findings were obtained with mechanical simulations [12, 17, 33, 38]. Galbusera et al. [17] revealed no differences on rotational stiffness between the bladed and other two shoe models with rounded studs. Thus, could be exaggerated to suggest that athletes must reject the bladed models, since they do not seem to increase the risk of noncontact injury [17]. However, because the material(s) used to construct the upper part of the shoe may influence rotational stiffness, future studies should explore this hypothesis [12].

Like in performance, the kind of grass also influences the risk of injury. When peak torque and the rotational stiffness was assessed by a mechanical instrument in different fields, the lowest peak torque was related to natural fields compared to four different artificial fields [38]. In addition, it has been argued that the grounds seem to be more important than the cleats in traction, linking again, the artificial grass to a higher risk of injury [8, 12].

In the future, it will be important to assess functional tasks and variables related to specific injuries in populations with higher risk, such as athletes with chronic ankle instability. Future studies involving jump strategies associated with different clinical conditions, like chronical ankle instability, are required, since the landing mechanism is a moment where a lot of injuries happen [36]. If possible, the fatigue protocols imposed to athletes should be closer to the reality of the game [37]. The methodological quality of studies in this area should also continue to be improved.

Globally, the mentioned studies highlight the TF as a protective model and the SG as a potentially harmful model for repetitive impact lesions, mainly in artificial fields. This is valid both in young [29] and adult players [16, 30]. When comparing the studs' geometry of the round aluminum studs and the bladed ones, the second model seems to boost the injury risk from the lateral border of the plantar surface [21]. It is still important to note that when comparing two fields (natural vs artificial), the second appears to potentiate injuries due to their rigidity [12, 38].

## 5. Conclusion

Cleat-surface interaction is an important and current topic, not only because it interferes with one of the soccer players' concerns (performance) but also with the injury risk and absenteeism from sport practice. Literature reveals a decreased sports performance with the SG model, a protective feature of the TF model cleat, and an increased risk of injury in the artificial grass. However, the health promotion literature continues to be slightly specific. The study of this interaction in healthy subjects under fatigue is essential, but very little has been studied so far. Also, because soccer player present a high prevalence of ankle sprains, the cleat-surface interaction should be evaluated in athletes with increased risk of ankle sprain, such those with chronic ankle instability. Finally, another important factor is the introduction of dynamic and unpredictable test protocols for the detection of differences in the cleat-surface interaction. The study of this interaction in the injury risk is an exciting field, but there is still much to explore. The results obtained about this topic will help sports health professionals to work more efficiently on injury prevention with the sports community.

## Figures and Tables

**Table 1:** Cleats' characteristics.

Cleat model		Indicated field	Studs/sole material	Studs		
				Number	Size	Geometry
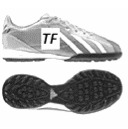	Turf	Synthetic	Rubber studs and compliant sole	>55	6-7 mm	Cylindrical, conical (rounded), prismatic, and bladed
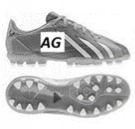	Artificial grass		Plastic studs and rigid plastic sole	22	8–10 mm	
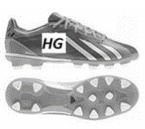	Hard ground	Dirt field		14	10–12 mm	
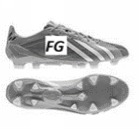	Firm ground	Natural ground in good conditions		11	10–12 mm	
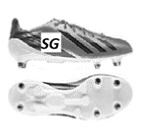	Soft ground	Muddy or wet natural ground	Aluminum studs and rigid plastic sole	6	13–16 mm	

**Table 2:** Number of papers collected from different databases.

Search terms	Scopus	Web of science	PubMed	B-on
Soccer shoes	66	44	34	59
Soccer boots				
Soccer cleats				
Soccer studs				
Soccer footwear				
Shoe-surface interface				
Shoe-surface interaction				

**Table 3:** Studies regarding the cleat-surface interaction on performance.

Author	Sample	Ground and cleat type	Methods and instruments	Variables	Conclusions	Quality index score (%)
Sterzing and Hennig [26]	20 male experienced soccer players: 25.4 ± 3.3 years 75.1 ± 7.1 Kg 177.6 ± 5.3 cm	*Ground*: (i) Artificial grass *Footwear condition:* (i) FG (0%, 50%,100% stud length) (ii) SG (100% stud length) (iii) Own soccer shoe (iv) Two premium cleat models	*Tasks*: 6 maximum kicks per shoe condition *Instruments*: (i) Stalker Pro radar gun (ii) Force platform	(i) Peak ball velocity (ii) erceived ball velocity (iii) Peak resultant shear force of the stance leg	Traction in the standing limb is partly influenced by the stud height, which in turn influences the kicking movement and ball velocity. The shoe weight and outsole stiffness had any effect on resultant ball velocity. Different shoe models alter the resultant ball velocity.	56,25%

Sterzing et al. [11]	52 male amateur or subelite soccer players: 24.5 ± 4.2 years 73.2 ± 7.0 Kg 177.9 ± 4.8 cm divided by 8 studies	*Ground*: (i) Dry and wet artificial grass (ii) Dry and snow natural grass *Footwear condition*: (i) HG (ii) FG rounded and bladed (iii) SG (iv) FG (0% stud length) (v) FG (50% stud length)	*Tasks:* (i) Straight line sprints (ii) Slalom *Instruments*: (i) Photovoltaic cells	(i) Running time (ii) Running time perception (given by a cleats ranking)	SG cleats with high studs' worse performance in synthetic. The behavior of the cleats on dry and wet ground was similar. Bladed studs improve performance compared with the elliptical ones in the slalom test, under dry and ice/snow conditions. Performance was gradually reduced with the reduction of stud height (50% and 0% of its original size). The increase of 70 g in shoes and the heel contour comfort does not seem to interfere with performance. The model defined by the manufacturers as indicated for synthetic turf favored performance compared to design model for natural grass, and it was perceived by athletes.	59,38%

Hennig et al. [27]	(i) 1st study—24 male subjects (ii) 2nd study—20 male subjects	*Ground*: (i) Not mentioned *Footwear condition*: 1st study—five different shoe modifications 2nd study—two shoes with significantly different accuracy in the 1st study	*Tasks*: (i) 20 repetitive inside and instep kicks towards a target *Instruments*: (i) Circular electronic target (ii) Plantar pressure insoles	(i) Mean ball deviation (cm) from the target (ii) Pressure distribution pattern	Although most soccer players are not aware of it, kicking speed and accuracy can be influenced by footwear design.	31,25%

Clarke and Carré [13]	A mechanical testing device was used instead of a soccer player sample.	*Ground*: (i) Artificial grass (ii) Natural grass *Footwear conditions*: (i) Six models of cleats with different studs' dimensions and geometry	*Tasks*: (i) Three trials of simulated sprints start*Instruments*: (i) Mechanical testing device with hydraulic system	(i)Penetration capacity (ii) Horizontal traction force	In natural grass, only highest and cylindrical studs do not fully penetrate, which may explain the lack of traction. The conical studs demonstrate better penetration. The average horizontal traction increases with the studs' cross-sectional area, but the opposite happens if the stud does not fully penetrate. Comparing two models of conical studs, in artificial turf, the lowest showed higher traction.	59,38%

Muller et al. [15]	25 male subelite soccer players:22.9 ± 4.1 years 71.5 ± 6.3 Kg 177.9 ± 4 cm A mechanical device was used to simulate sprint starts.	*Ground*: (i) Artificial grass *Footwear conditions*: (i) HG (ii) FG (iii) SG (iv) Prototype	*Tasks*: (i) Straight line sprints (ii) Slalom (iii) 45° and 180° changes of direction (iv) Simulation of sprints starts *Instruments*: (i) Photovoltaic cells (ii) Force platform (iii) Mechanical traction device	(i) Running time and their perception (ii) Peak vertical force (iii) Vertical force rate (iv) Peak shear force (v) Shear force rate (vi) Coefficient of traction	Athletes present worse performance with SG model compared to other models. The SG seems unable to fully penetrate into the artificial grass causing instability mechanisms.	56,25%

Sterzing et al. [14]	47 male experienced soccer players: 23.0 ± 3.2 years 71.4 ± 5.9 Kg 177.3 ± 4.4 cm divided into 3 phases of the study	*Ground*: (i) Artificial grass *Footwear conditions*: (i) HG (ii) FG (iii) SG (iv) Prototype (v) 4 variations of prototype	*Tasks:* (i) Straight line sprints (ii) Slalom (iii) 45° and 180° changes of direction *Instruments:* (i) Photovoltaic cells (ii) Force platform	(i) Running time and their perception (ii) Traction suitability perception (iii) Peak vertical force (iv) Peak a-p and m-l shear force (v) Peak resultant shear force (vi) Perceived ratios	The sole of the cleat developed was proved to be more suitable for synthetic compared to the 3 already commercialized models. Shoes with high studs (SG) do not seem to be the most suitable for artificial grass.	71,88%

Sterzing et al. [28]	19 male experienced soccer players: 24.0 ± 3.6 years 72.1 ± 3.1 Kg 178.3 ± 1.9 cm	*Ground*: (i) Artificial grass *Footwear conditions*: (i) FG rounded (ii) FG bladed	*Tasks*: (i) Dribbling (ii) One touch passes of rolling balls (iii) Lofted passes (iv) Reception passes (v) Passes from aerial (vi) Juggling *Instruments*: (1) 0–10 mm scale for handling suitability perception	(i) Ball handling suitability (ii) Dribbling (time and ball contacts) (iii) Juggling (ball contacts) (iv) Passes (cm)	Dribbling: FG bladed showed faster dribbling times compared to FG rounded model. Passes: no differences were found between footwear conditions.	59,38%

McGhie and Ettema [18]	22 male soccer players: 23.1 ± 2.8 years 77.5 ± 6.0 Kg 1.81 ± 0.1 meters	*Ground*: (i) 3 different artificial grass field heights: (42; 50; 60 mm) *Footwear conditions*: (i) TF (ii) FG rounded (iii) FG bladed	*Tasks*: (i) 5 short sprints with a 90°change of direction *Instruments*: (i) Force platform (ii) Motion capture system (iii) Photovoltaic cameras	(i) Peak impact (ii) Traction (iii) Total change in velocity (iv) Sliding velocity traction coefficient	The traction coefficient appears to be homogeneous as regards different combinations of cleats-ground, suggesting that individuals can adjust the gesture so as to maintain the desired level of traction in the task of changing direction.	71,88%

De Clercq et al. [25]	12 male soccer players: 16 ± 1 years 67.3 ± 8.1 Kg 1.76 ± 8.8 meters	*Ground*: (i) Dry and wet artificial grass *Footwear conditions*: (i) TF (ii) AG (iii) FG	*Tasks*: (i) 10 × 5 shuttle run tests with a 180°change of direction *Instruments*: (i) Force platform (ii) Perception of performance questionnaire	(i) Traction (ii) Time to finish the shuttle run test (iii) Players perception	Players perceived small differences in performance and traction. On dry artificial grass, the three tested stud designs do not affect performance or traction. On wet condition, the TF showed a larger shuttle run time. AG and FG fulfil the traction needs in both conditions.	62,50%

**Table 4:** Studies regarding the shoe-surface interaction on injury risk.

Author	Sample	Ground and cleat type	Methods and instruments	Variables	Conclusions	Quality index score (%)
Walter and Ng [29]	36 male children: 8 to 11 years	*Ground*: (i) Not specified *Footwear condition*: (i) Cleats with studs (ii) Cleats without studs	*Tasks*: (i) A straight run *Instruments*: (i) Plantar pressure insoles (ii) High-speed video	(i) Length of time from heel strike to heel lift (ii) Ankle dorsiflexion angle (iii) Plantar pressure distribution	The use of cleats with studs imposes a significant increase in dorsiflexion, which increases pressure on the growth center of the calcaneus. The high incidence of calcaneal apophysitis and the use of shoes with cleats in young populations might be related.	34,38%

Smith et al. [30]	6 male soccer players: 25 ± 4.18 years 79.7 ± 9.32 Kg	*Ground*: (i) Natural grass *Footwear condition*: (i) TF (ii) SG	*Tasks*: (i) A straight line slow (4,4 m/s) and fast running (5,4 m/s) *Instruments*: (i) Force platform	(i) Impact peak and loading rate (ii) Maximal breaking and propulsion forces (iii) Maximal medial and lateral forces	The aluminum cleats impose increased vertical forces and loading rates being consequently probably more associated with repeated impact injuries. Its use in hard grounds seems to be not advised.	43,75%

Livesay et al. [38]	A mechanical testing device was used instead of a soccer player sample.	*Ground*: (i) Natural grass (ii) 4 different artificial grass fields *Footwear condition*: (i) TF (ii) FG	*Tasks*: (i) Mimic a change of direction maneuver under a compressive load of 333 N *Instruments*: (i) Mechanical testing device	(i) Peak torque (ii) Rotational stiffness	The highest peak torques were developed by the FG model on the FieldTurf tray and by the TF model on Astroturf field combinations. The lowest peak torques were developed on natural grass field.	65,63%

Kaila [31]	15 male soccer players: 19.5 ± 1.4 years 70.1 ± 7.6 Kg 1.76 ± 0.06 meters	*Ground*: (i) Artificial grass *Footwear condition*: (i) 2 FG rounded (ii) 2 FG bladed	*Tasks*: (i) Straight-ahead run (ii) 30° and 60° sidestep cutting *Instruments*: (i) Motion capture system (ii) Force platform	(i) Internal/external tibia moments (ii) Valgus/varus moments (iii) Anterior/posterior joint forces (iv) Knee flexion angles (v) Vertical ground reaction forces	Different cleat type showed no difference on knee loading for each maneuver.	68,75%

Gehring et al. [32]	6 male soccer players: 25.2 ± 1.4 years 77.8 ± 8.3 Kg 183.2 ± 3.4 cm	*Ground*: (i) Artificial grass *Footwear condition*: (i) FG rounded (ii) FG bladed	*Tasks*: (i) A 180° turning movement *Instruments*: (i) Motion capture system (ii) Force platform (iii) EMG recorder	(i) Maximum ground reaction force (Fz, Fx, Fy) (ii) Peak EMG activity (quadriceps/hamstrings) (iii) Knee joint moments (flexion/extension)	Round and bladed studs showed no differences in externally applied knee joint loads. Higher activation of quadriceps femoris with round studs was showed during initial phase of stance.	62,50%

Queen et al. [16]	36 soccer players: 20.83 ± 3.05 years 71.12 ± 10.38 Kg 1.712 ± 0.082 meters (19 males and 17 females)	*Ground*: (i) Artificial grass *Footwear condition*: (i) TF (ii) HG (iii) FG rounded (iv) FG bladed	*Tasks*: (i) Running with side cut (ii) Change of direction of 180° *Instruments*: (i) Plantar pressure insoles	(i) Total time contact (ii) The contact area (iii) Maximum strength (iv) Peak pressure (v) Force time integral of the medial region, middle and side of the forefoot	In changing the direction of 180° and run with side cut, the foot peak pressure was significantly lower with the TF model compared with all others in both gender. Force time integral of the lateral forefoot region was higher on the bladed model, compared to the TF model in the males. In males, the total area of contact was significantly lower in the FG model compared to the TF model. In females, the force time integral and the medial forefoot maximum force was significantly lower with the TF model compared to all others.	78,13%

Villwock et al. [12]	A mechanical testing device was used instead of a soccer player sample.	*Ground*: (i) 2 natural grass (ii) 2 artificial grass *Footwear condition*: (i) 10 different models: (ii) 4 (rounded studs) (iii) 3 (bladed studs) (iv) 2 (replaceable studs) (v) 1 TF	*Tasks*: (i) Mobile testing apparatus was used to apply rotations at the shoe-surface interface. *Instruments*: mechanical testing device	(i) Maximum torque (ii) Rotational stiffness	Artificial grass fields showed increased rotational traction compared to natural grass which may lead to higher risk of injury. Maximum torque and rotational stiffness were not influenced by the studs' pattern. More malleable construction of the upper shoe can allow greater pronation during leg internal rotation. This can increase the probability of tibioperoneal rupture.	71,88%

Stefanyshyn et al. [33]	12 soccer players: 26.4 ± 6.2 years 74.0 ± 7.4 Kg 176.4 ± 4.1 cm A mechanical testing device was also used.	*Ground*: (i) Artificial grass *Footwear condition*: (i) Running shoe (ii) FG rounded (iii) SG rounded (iv) SG bladed	*Tasks*: (i) Cutting and turning movements at 4.0 ms^−1^ (ii) Translational traction (iii) Rotational traction *Instruments*: (i) Mechanical testing device (ii) Force platform (iii) Motion capture system	(i) Ankle joint moments: plantar/flexion; external rotation; eversion (ii) Knee joint moments: extension; external rotation; abduction (iii) Translational (iv) Rotational traction	Cutting movement: no significant differences in resultant ankle and knee joint moments between the shoe conditions. Turning movement: the FG (round), SG (round), and SG (bladed) had higher ankle and knee rotation moments than the running shoe. An increased rotational traction increases ankle and knee joint loading which in turn could potentiate a higher incidence of injury.	56,25%

Müller et al. [34]	15 soccer players: 20.7 ± 2.8 years 71.6 ± 5.4 Kg 176.3 ± 5.6 cm	*Ground*: (i) Artificial grass *Footwear condition*: (i) Cleat with studs completely removed (ii) Prototype (iii) FG (iv) SG	*Tasks*: (i) 135° turning movement *Instruments*: (i) Motion capture system (ii) Force platform	(i) Peak force (Fz, a-p) (ii) Foot angles (iii) Shank angles (iv) Foot translation (v) Maximum ankle and knee moments	Movement patterns for turning in different cleats were influenced by stud configuration and were primary found in the distal part of the lower extremities. Soccer players showed reduced mediolateral foot translation and increased ankle moments due to high and unsuitable traction. Cleats with studs completely removed (low traction) lead to movement adaptations in response to an increased risk of slipping.	62,50%

Bentley et al. [21]	29 male amateur soccer players Without anthropometric data of the sample	*Ground*: (i) Artificial grass *Footwear condition*: (i) SG rounded (ii) SG bladed	*Tasks*: (i) Straight run (ii) Slalom *Instruments*: (i) Plantar pressure insoles	(i) Peak pressure (ii) Pressure–time integral over 11 clinically relevant areas of the foot	The model with rounded studs can be considered more secure since it features normal pressure distributions while the model with bladed studs is potentially more harmful once it reveals increased pressures on the lateral border of the foot.	68,75%

Galbusera et al. [17]	A mechanical testing device was used instead of a soccer player sample.	*Ground*: (i) Artificial grass (ii) Natural grass *Footwear condition*: (i) FG rounded (ii) FG bladed (iii) SG rounded	*Tasks*: (i) Static preload of 1000 N and a rotation speed of 45°s^−1^ until a rotation of 140° was reached *Instruments*: Mechanical testing device	(i) Peak torque (ii) Rotational stiffness	Stiffness values were smaller on natural compared to synthetic field. No differences were found between models with bladed studs and those with rounded studs. This study does not confirm the hypothesis that blade-shaped cleats may be more associated with increased risk of noncontact injuries.	65,63%

Brock et al. [35]	14 soccer players: 20.1 ± 1.4 years 85.6 ± 9,7 Kg 1.81 ± 0.04 meters	*Ground*: (i) Artificial grass *Footwear condition*: (i) Running shoe (ii) Cleats with artificial grass studs (iii) Cleats with natural studs	*Tasks*: (i) 180° cut (ii) Single-leg 90° land cut *Instruments*: (i) Motion capture system (ii) Force platform	(i) Peak vertical and medial ground reaction forces (ii) Vertical loading rate (iii) Ankle and knee kinematic (range of movement, peak velocity, and peak angle)	Few differences in ground reaction forces or kinematic variables were observed between the shoe conditions. However, during 180° cut movement, natural grass studs produced the lowest peak medial ground reaction forces compared to other two models.	81,25%

Butler et al. [36]	28 soccer players (i) 14 males: 22.1 ± 3.9 years 73.3 ± 11.5 Kg 1.77 ± 0.66 meters (ii) 14 females 22.8 ± 3.1 years 64.4 ± 9.2 Kg 1.68 ± 0.07 meters	*Ground*: (i) Artificial grass *Footwear condition*: (i) Running shoe (ii) TF (iii) FG bladed	*Tasks*: (i) Header of a ball *Instruments*: (i) Motion capture system (ii) Force platform	(i) Peak dorsiflexion angle (ii) Peak plantarflexion moment (iii) Peak knee flexion angle (iv) Peak knee extension moment (v) Peak hip flexion and extension moment (vi) Peak vertical ground reaction force	Male soccer players exhibited an increased dorsiflexion with the bladed cleat compared to the running shoes or TF model. Female soccer players exhibited a reduction in peak knee flexion with the bladed cleat condition. The more rigid shoes seem to impair the female reception mechanism.	81,25%

Silva et al. [37]	28 male soccer players without ankle sprain history: 23.13 ± 1.9 years 68.36 ± 5.20 Kg 1.76 ± 0.06 meters All players presented *pes cavus*.	*Ground*: (i) Artificial grass *Footwear condition*: (i) TF (ii) HG (iii) FG	*Tasks*: (i) Five consecutive lateral jumps at a cadence of 120 beats per minute *Instruments*: (i) Pressure platform (ii) Force platforms (iii) Motion capture system (iv) EMG system (v) Isokinetic dynamometer	(i) Ankle eversion/inversion range of movement (ii) Loading rate of the vertical and lateral force (iii) Lateral and rearward displacement and speed of the COP (iv) Activation time of the long and short peroneals	In healthy soccer players, the contributor variables for ankle sprain were not influenced by the TF, HG, and FG cleats. The conclusions were similar even after an evertor-oriented fatigue protocol.	81,25%

## Additional Points

*Key Points*. (i) On artificial grass, the soft ground model is related to a decreased athlete's performance. (ii) On wet conditions, the turf model is related to decreased performance. (iii) The turf model provides higher protection against the risk of injury.
